# Chronic diarrhea in patients with acquired immunodeficiency syndrome (AIDS)

**DOI:** 10.1186/1471-2334-14-S4-P20

**Published:** 2014-05-29

**Authors:** Cristina Olariu, Adriana Nurciu

**Affiliations:** 1 Carol Davila University of Medicine and Pharmacy, Bucharest, Romania; 2 National Institute for Infectious Diseases “Prof. Dr. Matei Balş”, Bucharest, Romania; 3 University Emergency Hospital, Bucharest, Romania

## 

Chronic diarrhea, defined as two or more loose or watery stools per day for at least 1 month is common in patients with AIDS. It’s a causal relationship between the immunosuppression and the diarrhea in these patients. We evaluated the most common causes of diarrhea in patients with AIDS.

32 consecutive patients with AIDS were submitted with chronic diarrhea (19 males, 13 females), mean age 20.82±8.97 years. Generally, the same work up as for a patient without AIDS should be initiated: previous or recent history (including a review of the patient’s drugs), physical exam, blood tests, stool examination and abdominal ultrasonography were performed for all patients. Abdominal plain films, stool culture, total colonoscopy, upper endoscopy, CT (computed tomography) scanning or MRCP (magnetic resonance cholangiopancreatography) were done in selective cases.

Fifteen of the cases (46.87%) were with infectious enteritis (Cytomegalovirus or *Cryptosporidium*), *C. difficile*-associated diarrhea was observed in 5 cases (15.62%) and two patients (6.25%) were diagnosed with *Mycobacterium avium* complex infection. Four patients (12.5%) were diagnosed with inflammatory bowel diseases – 3 cases with ulcerative colitis and one patient with Crohn’s disease. For two patients (6.25%) the diagnosis was ascendant colonic adenocarcinoma and one patient (3.12%) was diagnosed with terminal ileum lymphoma. In 3 cases (9.37%) the final diagnosis was “AIDS enteropathy” (an enteric pathogen was not detected). However, there was a striking correlation between the severity of gastrointestinal diseases and the CD4 lymphocyte count.

Most common causes of chronic diarrhea in patients with AIDS are infectious enteritis (with CMV or *Cryptosporidium*) and *C. difficile*-associated diarrhea. Survival and outcomes are linked to severity of immunodeficiency.

